# The Government and the Voluntary Hospitals

**Published:** 1911-10-28

**Authors:** 


### The Hospital

The Workers' Newspaper of Administrative Medicine and Institutional Life, Administration, National Insurance and Health.

No. 1320, Vol. LI. SATURDAY, OCTOBER 28TH, 1911. PRICE ONE PENNY.

### THE GOVERNMENT AND THE VOLUNTARY HOSPITALS.

Now that Mr. Lloyd George, it is stated, has practically come to an agreement with the medical profession and friendly societies, we hope he may be able to give a little time and full consideration to the bearings of the Bill in relation to hospital provision and hospital benefit. The present position is one which no statesman who understands the issues involved can surely suffer to remain as it is at present. Under the Bill 15,000,000 insured persons will be created, but many of them will occupy a social position which in any case would render them ineligible for free medical relief under present conditions, and the whole of them, if the present constitution of the voluntary hospitals is to remain unchanged, will, as insured persons, not be fit objects for free hospital treatment after the passing of the Bill itself. Further, despite the fact that the insured will number 15,000,000 souls, or some 36 per cent. of the total population of the United Kingdom, the Bill will not touch a very large number of the poorer and more deserving members of the humbler classes, who have always needed adequate free hospital and medical care. With the exception of a relatively small number who are paupers, these humble folk are clearly fit objects for free medical relief in the voluntary hospitals.

Such being the case, it must become evident to every thoughtful person that the Bill as it stands, containing as it does no provision for hospital benefit, must at one and the same time create a great army of people who will require hospital treatment, that, they will not be eligible to receive and cannot obtain, if justice is to be done to the poor, at the voluntary hospitals. As insured persons they will be clearly ineligible for free medical relief. There will be, however, abundance of work for the voluntary hospitals if they take care of and provide for the humbler members of society, and the many women and children eligible, who are outside the Bill. In other words, the Bill as it stands, while it creates and recruits a vast army of insured persons, many of whom will need hospital care, contains no provision to meet the hospital requirements of this vast army of workers. Nearly, if not quite all, the available beds at the voluntary hospitals will in fact be required to provide for the pressing necessities and claims of the hundreds of thousands of poor entitled to free treatment who are not included in Mr. Lloyd George's scheme.

What, then, would be the position if the Bill went through without alteration and as it is at present? Either the insured who are seriously ill must be deprived of the hospital care without which they may suffer grievous injury and loss, or in the case of some of them, where successful pressure or interest may be brought to bear to secure admission to some voluntary hospital, they may receive free treatment at the cost of the humbler folk who are without the protection of the Insurance Bill, and for whose benefit the voluntary hospitals in fact were created. In any case, if the claims of the legitimate applicants for voluntary hospital care are to have the first consideration, there must be a large majority of insured persons without hospital provision of any kind.

In order to bring home clearly the way the Bill must affect the individual and the hospitals, so far as hospital treatment is concerned, we will take the case of London. The population of London, taking the Metropolitan and City police districts, in 1911 was upwards of 7¼ millions. Therefore we shall have created under the Bill, taking Mr. Lloyd George's figures, quite two and a half millions of insured persons in London, and probably more. According to the complete figures for the last year for which they are available, the number of in-patients treated to a termination in the wards of the voluntary hospitals, and the number of new out-patient cases treated at the voluntary and endowed hospitals in London, were quite two million persons. These figures show that the number of the insured persons in the Metropolis in a given year will in all probability be 20 per cent. greater than the present total hospital population. This is a striking fact to bear in mind, and it is one which will gain most force if the chairmen and secretaries in the provinces, who are organising the voluntary hospitals in their different centres, will work out the figures for each such centre, showing (1) the probable number of insured persons under the Bill, and (2) the actual hospital population in each such district to the latest date.

But this is not all. In dealing with urban communities so far as acute disease is concerned, to meet to a reasonable extent the claims of the population for hospital care, it is essential that the number of hospital beds shall be equal to at least 2 per 1,000 of the total population. On this basis the persons insured in London under Mr. Lloyd George's Bill will require in the ordinary course some 5,000 hospital beds to be exclusively set apart for their accommodation, or the needs of these persons for such relief cannot be reasonably met. To state these facts, which are demonstrably provable, is to prove that the Bill must be amended. This is so because, unless hospital benefit is provided for in the Bill, it must prove in practice a grave injustice to the insured, and an even graver injury in certain events to hundreds of thousands of the humbler classes who will be uninsured. This latter class will need in-patient and out-patient care, or they must perish; while the insured persons, unless the Bill is a failure, will secure medical treatment, at any rate, in their own homes. As the Bill stands at present, the more the question is examined, the more the relations of the voluntary hospitals to the Government and of the Government to the voluntary hospitals are considered, the more plainly it will appear that the time has arrived, when this urgent question of hospital provision for insured and uninsured people all over the United Kingdom must be faced on its merits, and dealt with in a statesmanlike and practical manner.

We hope very shortly to have for publication reports of the progress made in the formation of district hospital committees all over the country.

